# Formation and characterization of lipid droplets of the bovine corpus luteum

**DOI:** 10.1038/s41598-020-68091-2

**Published:** 2020-07-09

**Authors:** Heather A. Talbott, Michele R. Plewes, Crystal Krause, Xiaoying Hou, Pan Zhang, William B. Rizzo, Jennifer R. Wood, Andrea S. Cupp, John S. Davis

**Affiliations:** 1 Department of Obstetrics and Gynecology, Olson Center for Women’s Health, University of Nebraska Medical Center, Omaha, NE 68198-9450 USA; 2 Department of Biochemistry and Molecular Biology, University of Nebraska Medical Center, Omaha, NE 68198-5870 USA; 3 Veterans Affairs Nebraska-Western Iowa Health Care System, Omaha, NE 68105 USA; 4 Department of Pediatrics, University of Nebraska Medical Center, Omaha, NE 68198-5940 USA; 5 Department of Animal Science, University of Nebraska–Lincoln, Lincoln, NE 68583-0908 USA; 6 Present Address: Division of Reproductive and Developmental Sciences, Oregon Health Sciences University/Oregon National Primate Research Center, Beaverton, OR 97006 USA; 7 Present Address: Surgery Department, University of Nebraska Medical Center, Omaha, NE 68198-3280 USA

**Keywords:** Cell biology, Lipidomics, Lipids

## Abstract

Establishment and maintenance of pregnancy depends on progesterone synthesized by luteal tissue in the ovary. Our objective was to identify the characteristics of lipid droplets (LDs) in ovarian steroidogenic cells. We hypothesized that LDs are a major feature of steroidogenic luteal cells and store cholesteryl esters. Whole bovine tissues, isolated ovarian steroidogenic cells (granulosa, theca, small luteal, and large luteal), and isolated luteal LDs were assessed for LD content, LD-associated proteins and lipid analyses. Bovine luteal tissue contained abundant lipid droplets, LD-associated perilipins 2/3/5, hormone-sensitive lipase, and 1-acylglycerol-3-phosphate O-acyltransferase ABHD5. Luteal tissue was enriched in triglycerides (TGs) compared to other tissues, except for adipose tissue. Luteal cells were distinguishable from follicular cells by the presence of LDs, LD-associated proteins, and increased TGs. Furthermore, LDs from large luteal cells were numerous and small; whereas, LDs from small luteal cells were large and less numerous. Isolated LDs contained nearly all of the TGs and cholesteryl esters present in luteal tissue. Isolated luteal LDs were composed primarily of TG, with lesser amounts of cholesteryl esters, diglyceride and other phospholipids. Bovine luteal LDs are distinct from LDs in other bovine tissues, including follicular steroidogenic cells.

## Introduction

Luteal tissue forms in the ovary during each estrus or menstrual cycle and synthesizes progesterone, a steroid critical for early embryonic development and survival during pregnancy^1,2^. Luteal tissue has a tremendous ability to synthesize progesterone, secreting up to 40 mg/day in humans^3^, and even greater quantities in cattle^4^. The majority of the cholesterol utilized for progesterone biosynthesis in cattle comes from the blood in the form of high-density lipoprotein-derived cholesteryl esters with smaller amounts from low-density lipoprotein^5^. Lipoproteins are internalized either through receptor-mediated endocytosis or selective cellular uptake, where cholesterol is sorted from lipoproteins within endosomes^5^. Endosomal cholesterol is then believed to be trafficked to mitochondria for immediate progesterone biosynthesis or stored as cholesteryl esters in lipid reservoirs, also known as lipid droplets (LDs) for future steroid biosynthesis^5,6^. In addition to its vital role in mammalian fertility, progesterone is an essential precursor of androgens, estrogens, glucocorticoids and mineralocorticoids. Therefore, the high steroidogenic output of luteal tissue allows for detailed studies of steroidogenic mechanisms, which are likely conserved among steroidogenic cell types.

LDs store neutral lipids and are coated with LD-associated proteins that embed within the surrounding phospholipid monolayer. These LD-associated proteins stabilize the LD, interact with additional proteins that incorporate or remove lipids from the LD core, enable LD trafficking, and mediate association of LDs with other organelles^7^. The perilipin (PLIN) proteins, designated PLIN1-5, are a family of LD coat proteins that are important for stabilizing LD structure and provide a platform for protein assembly on the LD surface^7^. Although LDs have been observed in nearly all tissues, LDs have been most extensively studied in adipose cells, where they form large unilocular droplets^7^. In many circumstances, the formation of LDs is a sign of pathological conditions, such as the cholesteryl ester-laden foamy macrophage in atherosclerotic lesions^8^, fatty liver disease due to liver damage^9,10^, or adiposity due to storage of excess lipids^11^. However, in steroidogenic tissues, such as the ovary, LDs are a prominent and generally non-pathogenic feature—except in cases of genetic disorders of steroidogenesis^12^. Herein, we provide the first characterization of LD-associated proteins and lipases expressed in ovarian LDs.

Ovarian luteal tissue is easily distinguished from other tissues due to the abundant lipid content and cytoplasmic LDs. Luteal LDs exist in all species examined to date including: mice^13^, rats^14^, sheep^15^, cattle^16^, pigs^17^, buffalo^18^, rabbits^19^, bats^20^, non-human primates^21^, and humans^22^. The presence of LDs is used to distinguish active steroid-secreting luteal cells and luteal LDs are reported to contain both cholesterol and cholesteryl esters that can be used to synthesize steroids in both humans and cattle^23,24^. Strikingly, in rats and rabbits, luteal LDs are primarily composed of cholesteryl esters, and stimulation of steroid synthesis reduces LD cholesteryl ester concentrations^25^. Luteal LD presence and composition are known to be regulated by luteal trophic hormones, like luteinizing hormone, which decreases LD content^25^, luteolytic hormones, like prostaglandin F2α, that increase LD content^26^, and by diet^27^. With the knowledge of LD-associated proteins and lipids in other tissues, we set out to characterize LD features within the bovine luteal tissue, while it was actively secreting progesterone. Herein, we provide a comprehensive characterization of bovine luteal LDs. The present study compares luteal LDs to LDs in other tissues (adipose, hepatic, and cardiac) with differing metabolic profiles. This study provides the first characterization of LDs in large and small steroidogenic luteal cells, and provides a comprehensive analysis of the phospholipid and neutral lipid composition of luteal LDs.

## Results

### Visualization and quantification of luteal tissue LDs

**Figure 1:**
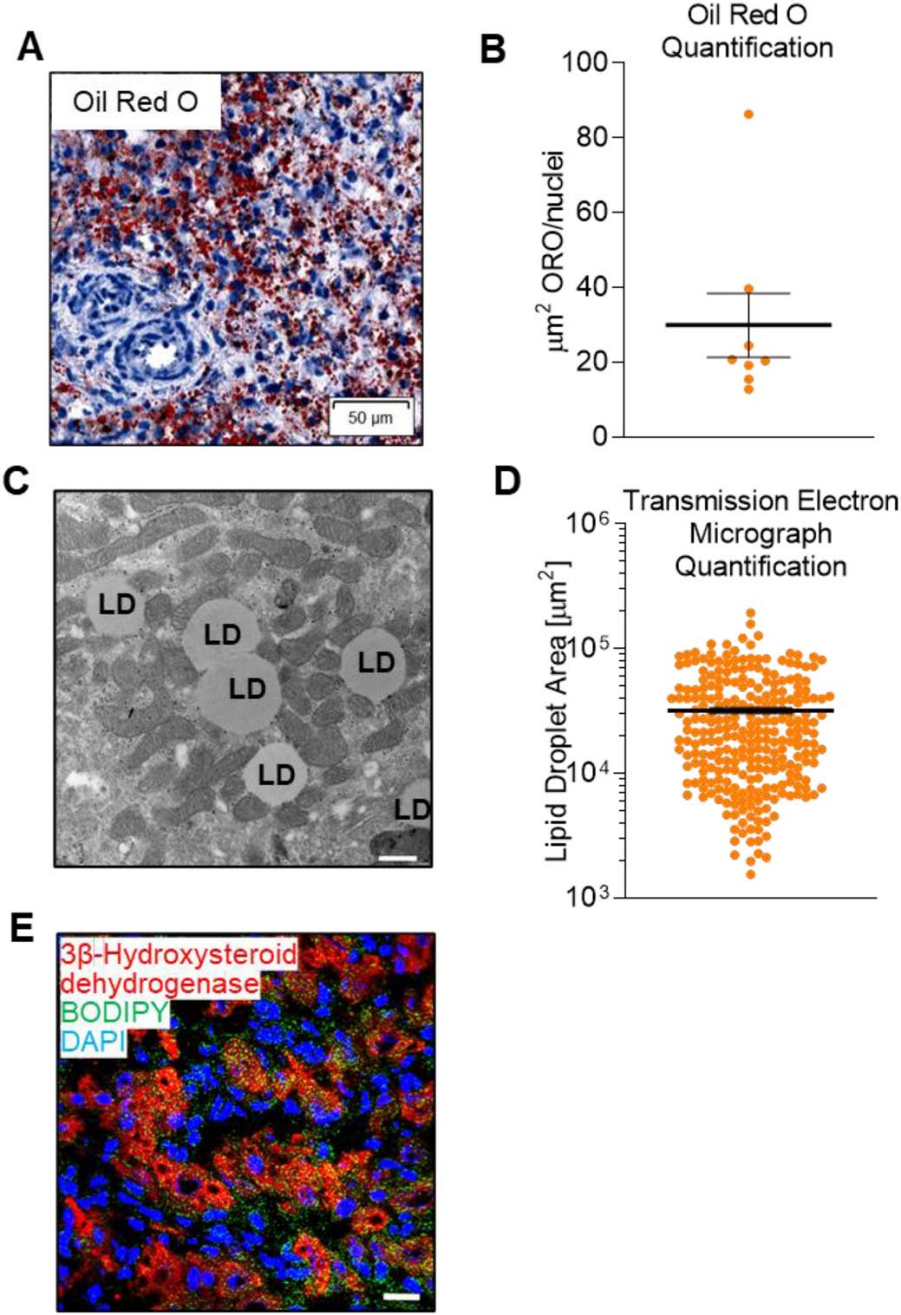
Visualization of luteal LDs. Luteal LDs were visualized using oil red O staining, transmission electron micrographs, and confocal microscopy. Panel (**A**) Oil red O staining (red) of lipids in frozen tissue sections of functional bovine corpus luteum counterstained with hematoxylin (blue). Representative image shown at 40×, scale bar = 50 µm. Panel (**B**) Automated quantification of tissue area occupied by oil red O staining, each point demonstrates the area (µm^2^) occupied by red immunohistochemistry staining in three randomly chosen images per animal (n = 6, mean ± SEM indicated with black lines). Panel (**C**) Transmission electron micrograph of bovine luteal tissue demonstrating multiple LDs (labeled LD) surrounded by mitochondria. Representative image shown at 21,000×, scale bar = 500 nm. Panel (**D**) Logarithmic graph of the quantification of area (µm^2^) occupied by individual LDs in three randomly chosen images per animal (n = 6, mean ± SEM are indicated with a black line). Panel (**E**) Representative confocal image obtained from frozen tissue sections of functional luteal tissue co-labeled with BODIPY493/503 (green; LD), HSD3B (red; marker for steroidogenic cells), and 4′,6-diamidino-2-phenylindole (DAPI, blue). Representative image shown at 63×, scale bar = 20 µm.

Functional bovine luteal tissue prominently featured LDs, which occupied the majority of luteal tissue, consistent with steroidogenic cell distribution within luteal tissue. However, vasculature and connective tissue invaginations within the luteal tissue (composed of non-steroidogenic endothelial and fibroblast cells) contained few LDs (Fig. 1A). Luteal LDs, stained with oil red O, occupied an average area of 29.9 ± 8.56 µm^2^ per nucleus within luteal tissue sections (Fig. 1A,B). Additionally, luteal LDs were an abundant ultrastructural feature of functional luteal tissue and were often in close proximity with mitochondria (Fig. 1C**)**. Individual LDs occupied an average area of 0.41 ± 0.04 µm^2^ in luteal tissue corresponding to an average diameter of ~ 0.72 µm and a range of 0.16–1.8 µm^2^ (Fig. 1C,D). Moreover, luteal LDs are present within the steroidogenic cells of luteal tissue, as confirmed by BODIPY-labeled LDs co-localized with a marker of steroidogenic cells, 3 beta-hydroxysteroid dehydrogenase/Delta 5– > 4-isomerase type 1 (Fig. 1E).

### Luteal tissue compared to other LD-associated tissues

**Figure 2:**
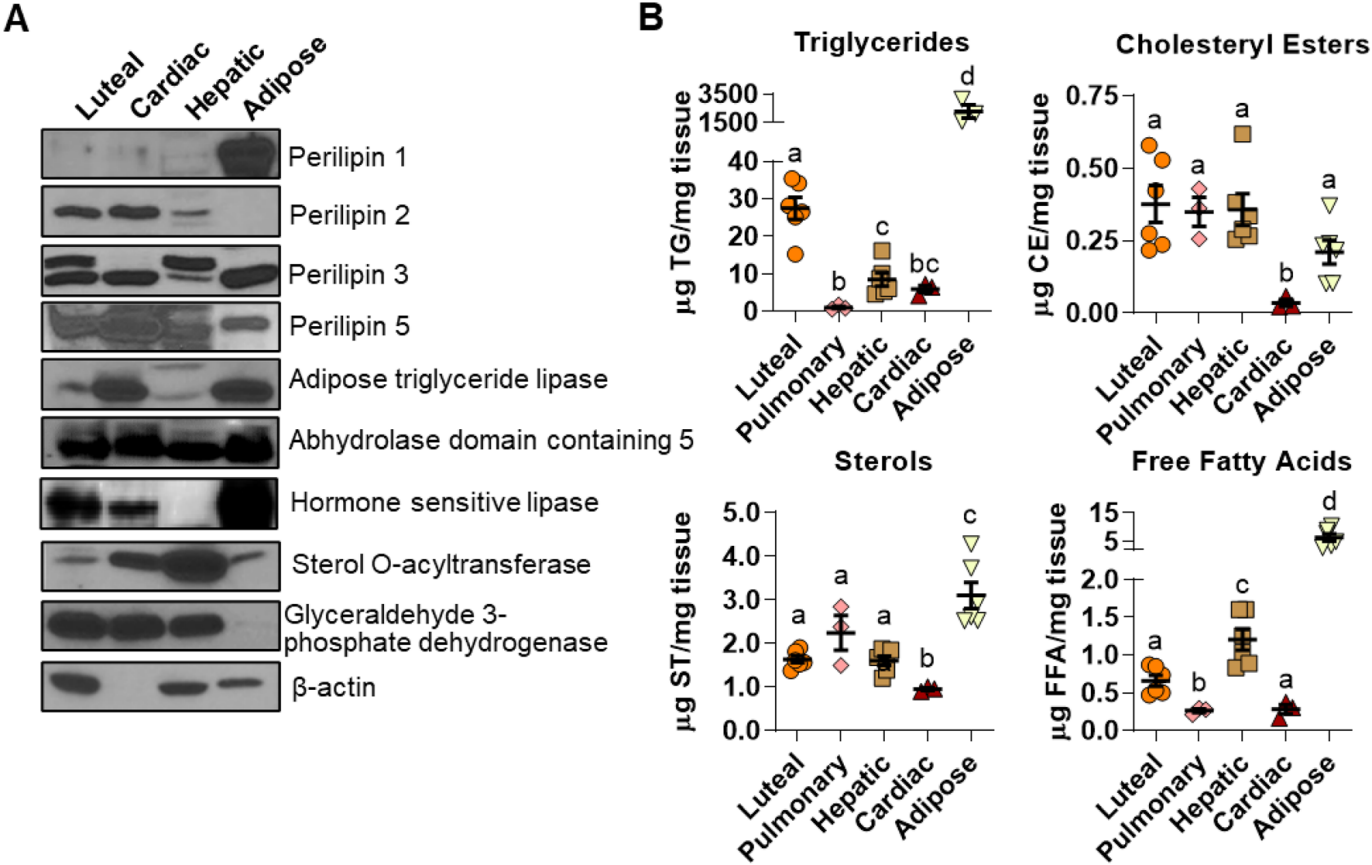
Lipid droplet (LD)-associated protein and lipid content of luteal tissue. Bovine luteal, cardiac, hepatic, pulmonary and visceral adipose tissues were collected to compare LD-associated protein and lipid content of luteal tissue between LD associated tissues. Panel (**A**) The presence of the LD-coat proteins (PLIN 1, 2, 3, and 5), key neutral lipid hydrolysis enzymes (ATGL, ABHD5, HSL), lipid forming enzymes (SOAT1) and loading controls (β-actin & GAPDH) were assessed by western blot. Panel (**B**) Lipid content of luteal tissue (n = 6) in comparison to pulmonary (n = 3), hepatic (n = 6), cardiac (n = 3), and visceral adipose tissue (n = 6) was determined by high-performance thin layer chromatography analysis to assess relative amounts of major neutral lipid classes. Horizontal line and error bars indicate mean ± SEM, significance was determined using mixed-effects model (factor 1: cell type (unmatched), factor 2: lipid class (matched)) after log transformation of values and Tukey’s multiple comparisons test. Means with different letters differ significantly between tissues (*P* < 0.05).

Bovine luteal tissue had a distinct composition of LD-associated proteins compared to other tissues (Fig. 2A). Luteal tissue contained considerable PLIN2 and PLIN5 protein, little to no PLIN1, and a moderate amount of PLIN3. In contrast, visceral adipose tissue had abundant PLIN1, hepatic tissue expressed PLIN2, and cardiac tissue had high amounts of PLIN2, PLIN3, and PLIN5. Bovine luteal tissue expressed substantial amounts of lipid-modifying enzymes: hormone sensitive lipase, 1-acylglycerol-3-phosphate O-acyltransferase abhydrolase domain containing 5 (ABHD5) , and lesser amounts of adipose triglyceride lipase (aka, patatin-like phospholipase domain-containing protein 2) and sterol O-acyltransferase 1. Hepatic tissue had the most sterol O-acyltransferase 1 and also expressed but had little hormone sensitive lipase and adipose triglyceride lipase. Adipose tissue had the most hormone sensitive lipase, large amounts of adipose triglyceride lipase, and less sterol O-acyltransferase than cardiac or hepatic tissue. Finally, cardiac tissue had large amounts of adipose triglyceride lipase, intermediate amounts of sterol O-acyltransferase, and a small amount of hormone sensitive lipase. Additionally, luteal tissue had a unique lipid composition, which is particularly evident in the high triglyceride (TG) content compared to pulmonary, hepatic, and cardiac tissue (Fig. 2B). Adipose tissue, as expected, had nearly 100-fold more TG than other tissues. Cholesteryl esters were a minor lipid class in all tissues investigated, and cholesteryl ester levels were nearly absent in bovine cardiac tissue. Sterol abundance was lowest in cardiac tissue and highest in adipose tissue; whereas, free fatty acids were highest in adipose tissue (fourfold), followed by hepatic tissue.

### Comparison of follicular and luteal cell type LD features

**Figure 3:**
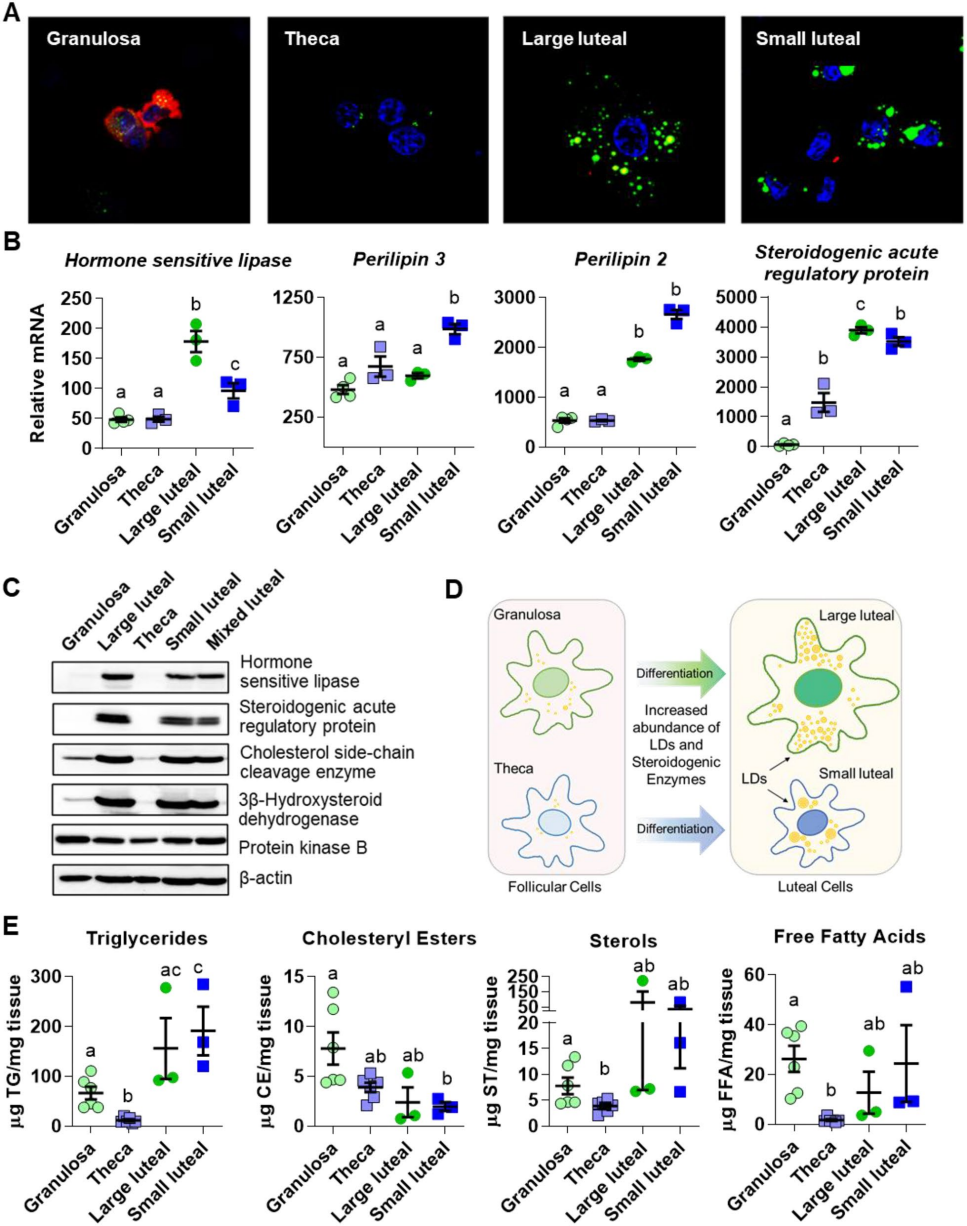
Comparison of follicular and luteal LD properties. Follicular granulosa cells, theca cells, large luteal cells, and small luteal cells were isolated from bovine ovaries as described in the Methods. Panel (**A**) Confocal fluorescent image showing LD staining in freshly isolated bovine granulosa, theca, large luteal, and small luteal cells. LDs were stained with the neutral lipid dye BODIPY 493/503 (green), and cells were immuno-labeled using an aromatase antibody to specifically label granulosa cells (red), and the nuclei are counter-stained with 4′,6-diamidino-2-phenylindole (DAPI, blue). All images are equal magnification. Panel (**B**) Microarray analysis of mRNA abundance of LD-coat proteins: perilipin 2 and 3, cholesteryl esterase: hormone sensitive lipase, and steroidogenic marker: steroidogenic acute regulatory protein. Significance determined in (34). Means with different letters differ significantly between cell types (*P* < 0.05). Panel (**C**) Western blot of LD-associated hormone sensitive lipase and steroidogenic enzymes in follicular granulosa and theca cells in comparison to large luteal, small luteal, and mixed luteal cells. Panel (**D**) Model depicting changes in LD number and proteins associated with luteal steroidogenesis during follicular cell differentiation. Panel (**E**) High-performance thin layer chromatography analysis of freshly isolated granulosa (n = 6), theca (n = 6), large luteal (n = 3) and small luteal cells (n = 3). Means ± SEM overlay individual measurements, significance was determined using mixed effects model and Tukey’s multiple comparisons test (factor 1: cell type (unmatched), factor 2: lipid class (matched)) after log transformation of values. Means with different letters differ significantly between cell types (*P* < 0.05).

Follicular granulosa and theca cells differentiate to give rise to large and small luteal cells after ovulation^28^. LD content was compared between the follicular and luteal cell types. Granulosa and theca cells had fewer and smaller LDs than the luteal cell types, as assessed by BODIPY staining of LDs and confocal imaging (Fig. 3A). Cytoplasmic staining for aromatase confirmed the identity of granulosa cells. A microarray comparison^29^ between granulosa, theca, large luteal cells and small luteal cells indicated that mRNA abundance of LD-associated proteins *PLIN2*, *PLIN3* and hormone sensitive lipase (*LIPE)* were increased in luteal cell types compared to follicular cells (Fig. 3B). Similarly, mixed luteal cells and both small and large luteal cells had greater amounts of hormone sensitive lipase compared to granulosa and theca cell isolates, along with greater expression of steroidogenic enzymes: Steroidogenic acute regulatory protein, cholesterol side-chain cleavage enzyme, and 3 beta-hydroxysteroid dehydrogenase/Delta 5– > 4-isomerase type 1 (Fig. 3C). These data indicate that during differentiation of ovarian follicular cells (granulosa and theca) into luteal cells (large and small luteal cells) there are parallel increases in LDs and steroidogenic enzymes (Fig. 3D). Additionally, granulosa cells contained more TG, sterols, and free fatty acids than theca cells. Moreover, large and small luteal cells had more TG than granulosa or theca cells, as determined by high-performance thin-layer chromatography (Fig. 3E).

**Figure 4:**
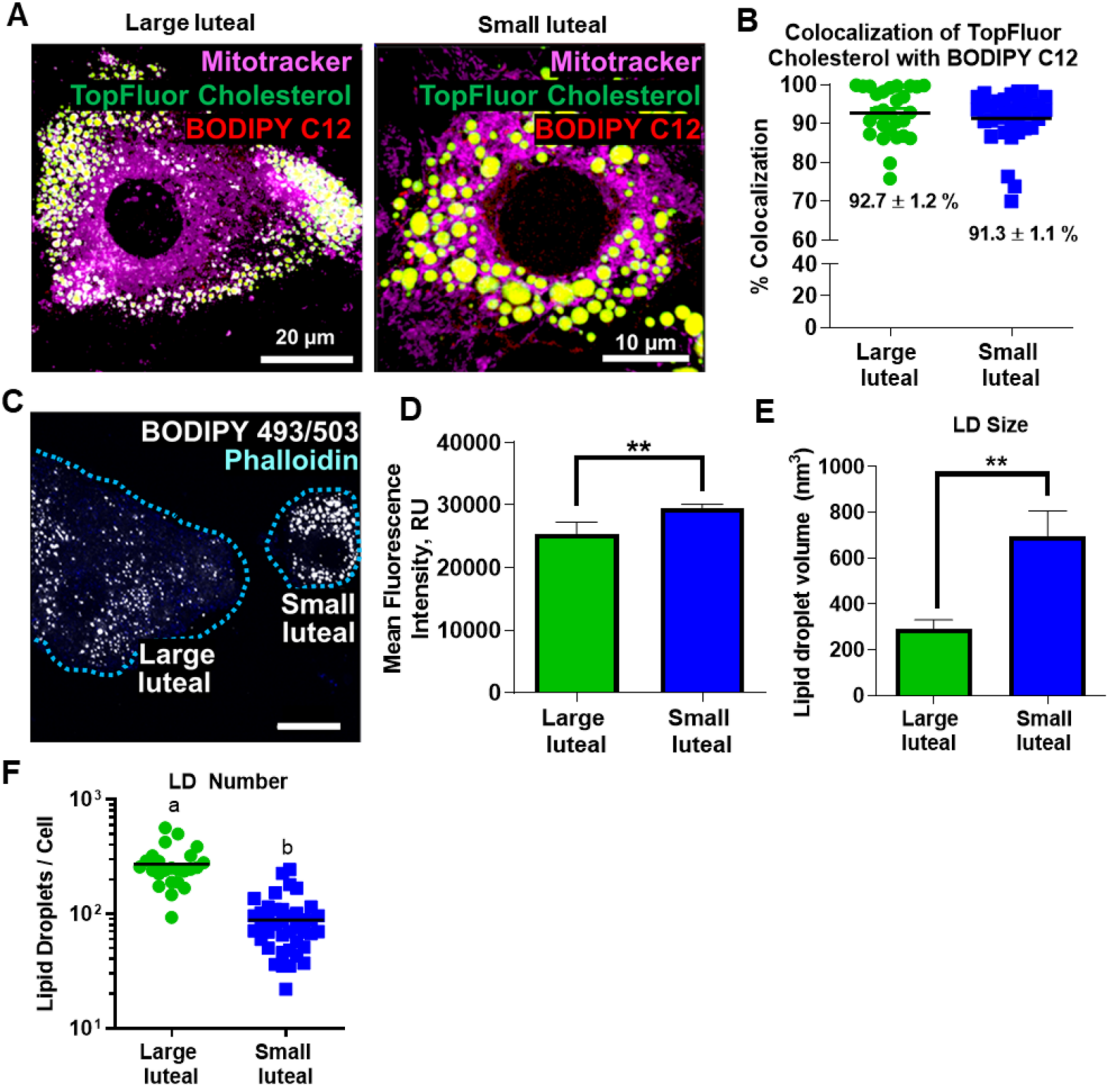
Differences in LDs between small and large luteal cells. Individual LD measurements in freshly isolated large luteal cells and small luteal cells were determined using confocal microscopy. Panel (**A**) Representative confocal micrographs of enriched large luteal and small luteal populations pre-loaded with BODIPY C12 and TopFluor Cholesterol for 48-h. Mitochondria (Mitotracker; magenta), TopFluor Cholesterol (green), and BODIPY C12 (red); Micron bars represent 20 and 10 µm, respectively. Panel (**B**) Quantitative analysis of percent colocalization of TopFluor Cholesterol with BODIPY C12 in large luteal cells (green) and small luteal cells (blue). Panel (**C**) Individual lipid droplet (LD) measurements in freshly isolated large and small luteal cells were determined after BODIPY staining of three-dimensional rendering of confocal images. Representative images of large luteal cells and small luteal cells. BODIPY 493/503 (white) and Phalloidin (blue); Dashed blue line indicates cell boundaries. Micron bar represents 20 µm. Panel (**D**) Quantitative analysis of mean fluorescence intensity for large luteal and small luteal cells. Significance of data was assessed with unpaired two-tailed t-test. Panel (**E**) Quantitative analysis of individual LD size, large luteal (25 individual cells), small luteal cells (41 individual cells) from 3 animals are displayed as mean ± SEM. Panel (**F**) Logarithmic graph displaying the quantitative analysis of number of LDs per cell, significance was assessed using unpaired two-tailed t-test after log transformation of the data.

Khor et al*.* suggest that cholesterol and TG can segregate into distinct LD populations^30^. Large luteal cell and small luteal cells both had substantial (> 90%) colocalization of fluorescently-labeled cholesterols and fatty acids within LDs, as examined by confocal microscopy (Fig. 4A,B). Large luteal cells had significantly more LDs (271 ± 22) compared to small luteal cells (89 ± 8) (Fig. 4C,E); whereas, small luteal cell LDs were significantly greater in volume (695.5 ± 112.3 nm^3^) than large luteal cell LDs (293.2 ± 39 nm^3^, Fig. 4B,C), visualized by confocal microscopy. The measured LD volumes correspond to an average LD diameter of 1.1 µM for small luteal cells and 0.82 µM for large luteal cells. In agreement with volume measurements, small luteal cells had more lipid content than large luteal cells as measured using mean fluorescence intensity to estimate the total lipid content (Fig. 4D,F).

### Composition of isolated luteal LDs

**Figure 5:**
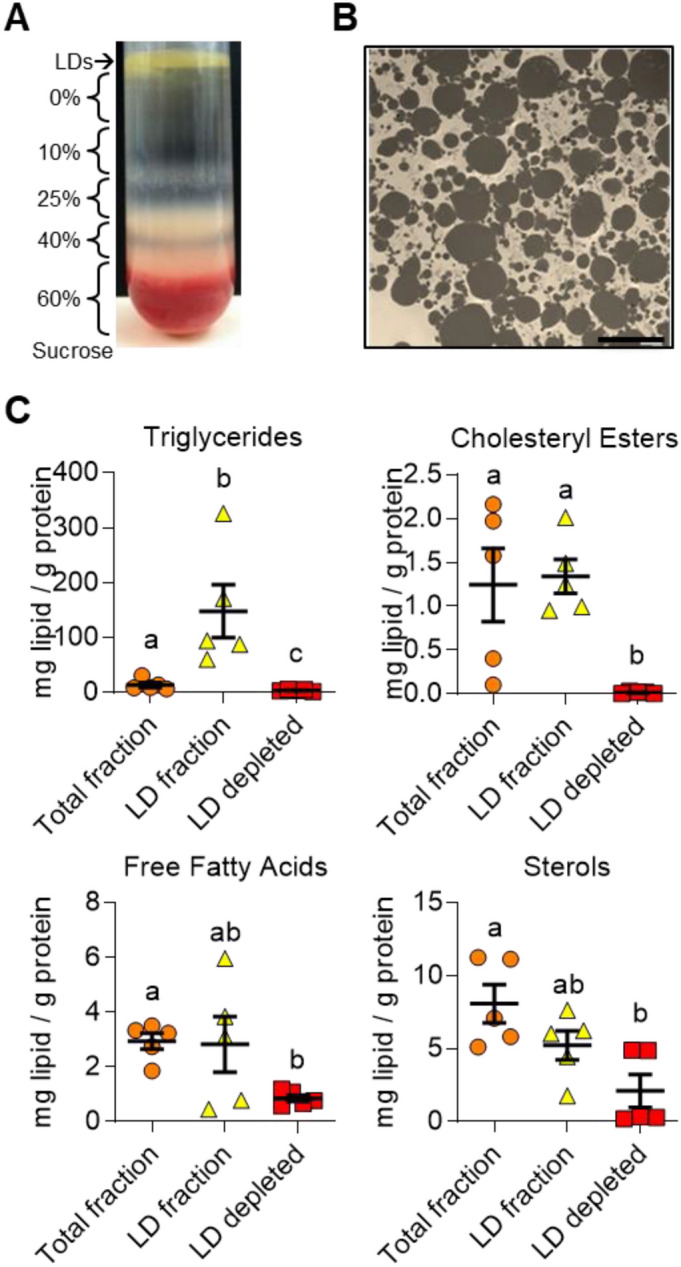
Isolated luteal LD properties. Sucrose gradient and ultra-centrifugation was used to isolate luteal LDs. Transmission electron micrography images were obtained to validate successful isolation of luteal LDs. Panel (**A**) Representative image of luteal LDs and sucrose gradient fractions following ultra-centrifugation. Panel (**B**) Representative transmission electron micrograph from isolated LD preparations. Micron bar represents 6 µm. Panel (**C**) Lipid analysis by high-performance thin layer chromatography of whole luteal tissue fraction (total), LD fraction, and LD depleted post-nuclear supernatant. Lipid content of each fraction was normalized to protein content. Bars represent means ± SEM, significance was determined using mixed-effects model and Tukey’s multiple comparison test (factor 1: subcellular fraction (unmatched), factor 2: lipid class (matched)) after log transformation of values, n = 5. Means with different letters differ significantly between tissues (*P* < 0.05).

Isolated luteal LDs, prepared by a step-wise sucrose gradient, were yellow in color in comparison to LDs from other tissues (Fig. 5A). Intact LDs, imaged by transmission electron microscopy, were relatively free from other cellular organelles and debris after isolation (Fig. 5B). The cellular localization of lipid classes in the total luteal tissue fraction, the isolated LD fraction, and the LD-depleted fraction were compared using high-performance thin layer chromatography (Fig. 5C). TGs were primarily stored in luteal LDs, with nearly no TG found in LD-depleted lysates. Similarly, cholesteryl esters were present in the LD fraction and absent in LD-depleted lysates. Sterols and free fatty acids showed little segregation between LDs and LD-depleted cellular fractions. Lipid extracts of LDs were characteristically yellow which deepened in color during concentration of LDs and of LD lipids. During high-performance thin layer chromatography a yellow band, presumably carotenes, consistently migrated along the solvent front with cholesteryl esters (not shown). Additionally, a band of unknown lipid species consistently migrated between the TG and cholesteryl ester standards when separated by high-performance thin layer chromatography (not shown), possibly ether-linked lipids, which have been reported in adiposome LDs^31^.

**Figure 6:**
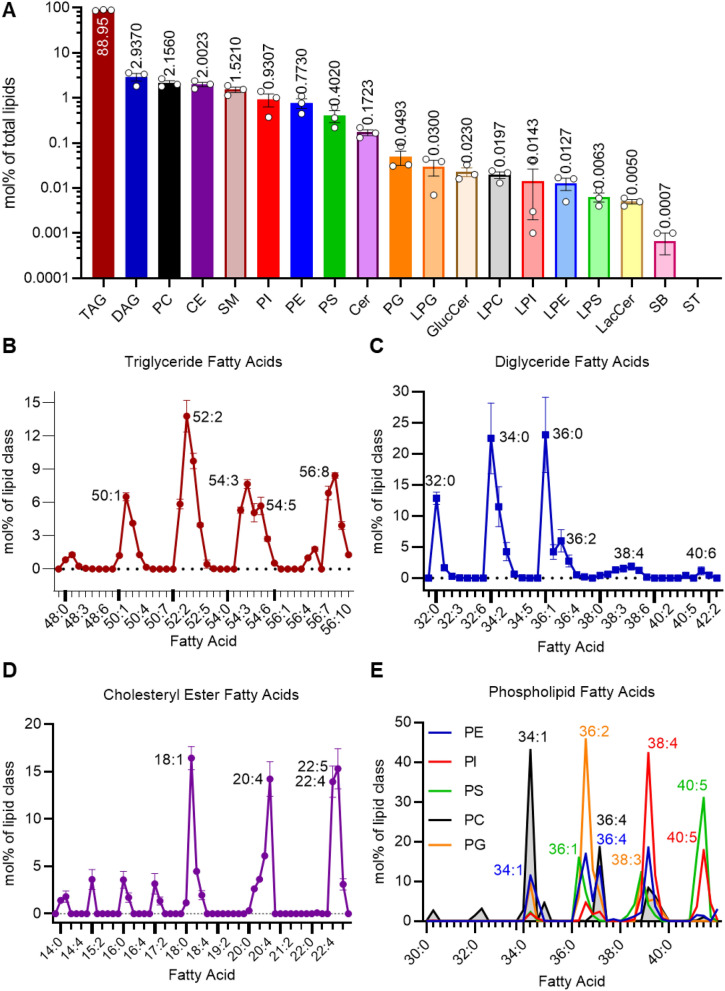
Lipidomic analysis of isolated luteal lipid droplets (LDs). LC/MS/MS with sMRM was used to detect lipid species with lipid class specific internal standards to allow for relative quantification. Panel (**A**) The top 18 lipid classes present in bovine luteal LDs are shown from the largest proportion of the total lipid fraction to the smallest as measured as the mol% of the specific lipid class to the total lipid content, means ± SEM overlay individual measurements, n = 3. Cholesteryl Ester (CE); Glucosylceramide (GlucCer); sphingoid bases (SB). Panel (**B**) The combined fatty acid composition of TG in luteal LDs are represented as the mol% within the TG class, with increasing degrees of saturation listed within the shortest to longest combined acyl chain length, peaks are labeled with the relevant fatty acid, points indicate means ± SEM. Panel (**C**) The fatty acid composition of the diglycerides in luteal LDs are represented as the mol% within the diglyceride class, points indicate means ± SEM. Panel (**D**) The combined fatty acid composition of the cholesteryl esters in luteal LDs are represented as the mol% within the cholesteryl ester class, points indicate means ± SEM. Panel (**E**) The fatty acid composition of several phospholipid classes (Phosphatidylethanolamines (PE), Phosphatidylinositol (PI), Phosphatidylserine (PS), Phosphatidylcholines (PC) and Phosphatidylglycerol (PG)) in luteal LDs are represented as the mol% within the appropriate lipid class, peaks are labeled with the relevant fatty acid, inflection points indicate means.

Lipidomics analysis of three different preparations of luteal tissue LDs confirmed that luteal LDs are primarily composed of TG (168 ± 42 pmol/µg protein, 89 mol% of total lipids) but also contain many other lipid classes (Fig. 6A). Other neutral lipids included diglycerides (DGs; 5.7 ± 2.1 pmol/µg protein, 2.9%); and cholesteryl esters (3.65 ± 0.7 pmol/µg protein, 2.0%). Polar lipids were primarily composed of phosphatidylcholines (PC; 3.9 ± 0.8 pmol/µg protein, 2.2%), sphingomyelins (2.7 ± 0.3 pmol/µg protein, 1.5%), phosphatidylinositols (1.7 ± 0.6 pmol/µg protein, 0.9%), phosphatidylethanolamines (1.4 ± 0.4 pmol/µg protein, 0.8%) and phosphatidylserines (0.65 ± 0.04 pmol/µg protein, 0.4%). A number of other minor lipids representing less than 0.33 mol% of the total lipid pool were also detected including phosphatidylglycerol, lysophosphatidylinositol, lysophosphatidylcholine, lysophosphatidylethanolamine, lysophosphatidylserine, ceramides, glucosylceramides, and sphingoid bases (Fig. 6A). Sterols were below the limit of detection (lowest concentration of standard curve) in all three LD samples. The polar to nonpolar lipid ratio was 7.12 ± 0.017, which corresponds to an average LD diameter of 282 nm if the properties of sphericity and surface area to volume ratios are used to calculate the expected LD diameter^32^.

Each lipid class was composed of a diversity of fatty acyl species. The TG class was represented by 28 species, of which 26 molecular species were found in all three samples (Fig. 6B). Four principal groups with 52, 56, 54, and 50 acyl carbons were detected, each with a number of double bonds. The predominant species was 52:2 (total acyl carbons:total acyl double bonds) representing ~ 14 mol% of the TG class; and the combination of TG(52:3), TG(56:8), TG(54:3), TG(56:7), and TG(50:1) species represented 39% of TGs. The DG class was represented by 30 molecular species (29 detected in all three samples) (Fig. 6C). Three principal DG groups with 32, 34 and 36 acyl carbons were identified, none of which contained double bonds. Approximately 58% of the DG pool was composed of the DG(32:0), DG(34:0), and DG(36:0) species. The cholesteryl ester class was composed of 20 molecular species (all species were detected in all samples) (Fig. 6D) with four principal cholesteryl ester-linked fatty acid moieties: (18:1), (20:4), (22:4) and (22:5) which made up 60% of cholesteryl esters.

The luteal LD phospholipid composition (expressed as a percentage of total phospholipid) was predominately PC (37%) followed by sphingomyelin (26%), phosphatidylinositol (15%), phosphatidylethanolamine (13%), phosphatidylserine (7%) and phosphatidylglycerol (0.8%), while lysophospholipids only accounted for 1.4% of phospholipids. The phospholipid composition of luteal LD was surprisingly complex with more than 120 molecular species (85 detected in all three samples) in five classes (Fig. 6E). Analysis detected a total of 31 PC molecular species (22 detected in all three samples), with PC(34:1) contributing to 43% of the population and an additional 33% of the PC class was composed of PC(36:4), PC(38:4), and PC(38:5). Lipidomics detected 34 species of phosphatidylethanolamines (27 detected in all samples) which were dominated by phosphatidylethanolamine-linked fatty acids: (38:4), (36:2), (36:4), and (34:1). Twenty species of phosphatidylinositols were detected (16 in all three samples) with 72% of the total phosphatidylinositol pool was represented by phosphatidylinositol-linked fatty acids: (38:4), (40:5), and (38:5). Lipidomics detected 18 species of phosphatidylserines (13 in all three samples) with 76% of the total pool represented by phosphatidylserine-linked fatty acids: (40:5), (40:4), (36:1), and (38:3). The phosphatidylglycerol class was composed of 14 species (7 detected in all samples) with phosphatidylglycerol (36:2) contributing to 46% of the total phosphatidylglycerol pool.

## Discussion

Luteal LDs are a prominent feature of bovine luteal tissue and the presence, prominence, and composition of luteal LDs distinguish luteal tissue and luteal cells from other tissues and cell types. The large and small steroidogenic luteal cells contain these distinguishing LDs; with large luteal cells containing numerous small LDs, and small luteal cells processing fewer, but larger, LDs. Luteal LDs have a diameter of 200–1,100 nm, confirmed by a variety of techniques including transmission electron microscopy, confocal microscopy, and phospholipid : total lipid ratio. Purified luteal LDs are rich in TG and contain cholesteryl esters, supporting the concept that luteal LDs have an important role in cellular metabolism and steroid biosynthesis by storing steroidogenic precursors.

Luteal tissue of all examined species contain abundant LDs, which vary in size and number throughout the normal estrous or menstrual cycle^14–18^. The findings that neutral lipids occupy approximately 5–16% of bovine luteal tissue area is in agreement with other studies examining LD to luteal cell area or volume^26,27,33^. In contrast, other tissues rarely contain the same extant of neutral lipids, except during pathological situations^8–11^. Likely, the area occupied by LDs in steroidogenic cells is even greater since luteal endothelial cells account for nearly 50% of total luteal cells^34^ but rarely contain LDs^35^. The present study and previous ultrastructural studies have noted the close contact of LDs with mitochondria^16,20,22^. The proximity of luteal LDs to mitochondria suggests that these organelles may function cooperatively to synthesize steroids^6,36^. The dynamics of LD and mitochondria interactions in luteal cells are unknown, but recent studies in other tissues implicate the mitochondrial targeting regions of PLIN5^37^ and diacylglycerol O-acyltransferase 2 can facilitate physical interactions between mitochondria and LDs^38^. The emerging field of mitochondrial-associated membranes offers the possibility that there are regionalized areas of contact between mitochondria, endoplasmic reticulum and LDs^39,40^.

The size, distribution, protein, and lipid content of LDs can differ among various tissues^7,36^; therefore, expression of LD-associated proteins in bovine luteal tissue was compared with cardiac, hepatic, and adipose tissues. The tissues differed in the relative composition of PLINs and other lipid modifying enzymes, consistent with previous reports for adipose^41^, cardiac^42^, and hepatic tissue^10^. Luteal tissue expressed PLIN2, PLIN3, PLIN5, hormone sensitive lipase, and 1-acylglycerol-3-phosphate O-acyltransferase ABHD5; and lower levels of adipose triglyceride lipase and sterol O-acyltransferase proteins, whereas, PLIN1 was undetectable. The presence of PLIN2, PLIN3, and PLIN5 suggest luteal LDs are metabolically active, oxidative, and mitochondrial-associated^7^. As well, bovine tissues differed in lipid composition. Bovine luteal tissue contained higher levels of TG than other tissues (cardiac, hepatic, & pulmonary) with the unsurprising exception of adipose tissue. The high TG content of luteal tissue has unknown importance but could be the result of re-esterification of fatty acids liberated from cholesteryl esters during steroidogenesis to prevent fatty acid induced lipotoxicity. The unique LD-associated protein and lipid content of luteal LDs suggests luteal LDs have specialized lipid use and storage needs compared to other tissues.

After ovulation, the granulosa and theca cells of the follicle differentiate into the large and small luteal cells of the corpus luteum^28,29^. In contrast to the preovulatory follicle, which contains few LDs^23,43^, luteal tissue possesses numerous LDs. In this study, luteal tissue had fully established luteal LD content by three days post-ovulation; consistent with the idea that ovulation induces the formation of LDs during differentiation of the ovarian follicle into luteal tissue. Additionally, both large luteal cells and small luteal cells have abundant LDs and contain more TG in comparison to either granulosa or theca cells. These observations are in keeping with studies by Guraya et al*.,* who described that following the luteinizing hormone surge, human granulosa cells develop fine “lipid granules” and “heterogeneous lipid bodies” within newly ruptured follicles, and that the theca interna cells of newly ruptured follicles fill with sudanophilic lipids, including cholesterol and cholesteryl esters^23^. As well, LD numbers and size increase in granulosa cells within a day after ovulation in rabbits^43^. Bovine luteal cells, in comparison to follicular cell types, have more LD-associated proteins, which parallel the increase in LD formation and steroidogenic capacity. Similarly, treatment of rhesus macaques with luteinizing hormone increases the amount of PLIN2 protein in granulosa cells within 12 h^21^. The program of cellular differentiation following ovulation resulting in luteinization of granulosa and theca cells is accompanied by increased expression of LD-associated proteins, elevation in TG, and formation of LDs in steroidogenic luteal cells.

The LDs of large and small luteal cells differ primarily in the number and size of LDs per cell, with minimal differences in lipid content and LD-associated mRNAs. Small luteal cells have a mean LD volume 2.4 times greater than large luteal cells; whereas, large luteal cells have three times the number of LDs present in small luteal cells. This finding is in agreement with data by Khanthusaeng et al*.*, who reported that ovine large luteal cells have many smaller LDs and small luteal cells have fewer, but larger LDs^33^. The difference in LD size and number in the two cell types may relate to the different functions of large and small luteal cells. Bovine large luteal cells are responsible for secretion of large quantities of progesterone under basal conditions, whereas, small luteal cells secrete less progesterone under basal conditions but respond acutely to luteinizing hormone with robust increases in progesterone production^44^. Small LDs in large luteal cells could provide increased accessibility for continuous substrate utilization, such as liberation of cholesterol for constitutive steroid synthesis^7^. The larger LDs seen in small luteal cells could function primarily as a lipid storage mechanism, which can be accessed as needed, such as in response to hormones that stimulate steroidogenesis^7^.

Cholesteryl esters and TGs are almost exclusively located in luteal LDs. Bovine luteal LDs contain primarily TG (89% of total lipid content), and are relatively cholesteryl ester-poor (3.7 ± 0.7 pmol/µg protein; 2% of total lipid), findings similar to whole bovine luteal tissue^24^. Although cholesteryl esters and TG can segregate into distinct LD populations^30^, the LDs in large or small luteal cells show little segregation of TG and cholesteryl esters, which may reflect species or cell type differences. In contrast to bovine luteal tissue, luteal tissue from rabbits and rats is cholesteryl ester-rich^25^. However, bovine luteal tissue does not rely on de novo cholesterol synthesis for progesterone synthesis^45^; therefore, a small pool of cholesteryl esters may be sufficient for luteal function. The high TG content in bovine luteal LDs may serve as a substrate for energy production to fuel the steroidogenic output of luteal tissue. The fatty acids derived from cholesteryl esters may be converted to biologically active lipid mediators, re-esterified and stored in LDs or cell membranes, or used for β-oxidation, ultimately producing acetyl-CoA for the citric acid cycle. It seems likely that the production of large quantities of progesterone by luteal cells could require β-oxidation of fatty acids to provide the energy needed for optimal steroidogenesis.

Mass spectrometry was used to obtain detailed information about the lipid composition of bovine luteal tissue LDs. LDs are composed of a core consisting of neutral lipids (TGs and cholesteryl esters) bound by a monolayer of phospholipids^7^. The phospholipid composition of isolated droplets is remarkably complex. Luteal LD phospholipid composition (expressed as a percentage of total phospholipid) of luteal LDs is predominantly PC (45%) followed by sphingomyelins (22%), phosphatidylinositols (13%) and phosphatidylethanolamines (11%). In contrast, phospholipid content of hepatic LD from fed animals was largely PC (61%) and phosphatidylethanolamines (23%) with lesser amounts of phosphatidylinositols and sphingomyelins. The present results also differ somewhat from a report on the phospholipid content of LDs isolated from various cells lines following incubation with oleate to induce LD formation; the LD were predominately PC rich followed by phosphatidylethanolamine and phosphatidylinositol, as noted above, but were deficient in sphingomyelins and phosphatidylserines^31,46,47^. When directly compared, luteal LDs had ~ 15-fold more sphingomyelins and ~ 8.5-fold more phosphatidylserines than either mouse hepatic tissue or CHO K2 cells^31,46,47^. Despite the tissue and cell type differences in lipid class abundance, analysis of the specific TG, DG, PC, phosphatidylethanolamine and phosphatidylinositol species revealed that the constituent fatty acids within each phospholipid class are similar to previous studies^31,46,47^.

In steroidogenic tissues, the hydrolysis of cholesterol esters by hormone sensitive lipase yields cholesterol and a free fatty acid. The cholesterol serves as a precursor for mitochondrial steroid synthesis^48^, and while the fate of the fatty acid is unknown, it could be converted to biologically active lipid mediators, re-esterified and stored in LDs or membranes, or used for energy production. The cholesteryl esters in bovine luteal LDs are primarily (60%) mono- and polyunsaturated fatty acids evenly distributed among oleic acid, and the omega-3 and omega-6 20:4, 22:4 and 22:5 fatty acids. This differs from ovine luteal cholesterol ester fatty acids which are predominantly palmitic acid [(16:0), 30.7%], oleic acid [(18:1) 22.3%] and linoleic acid [(18:2) 17.5%]^15^. Cholesteryl ester fatty acid content of rat luteal tissue had many similarities to those in bovine luteal LDs but contained more palmitic acid (16:0), and less oleic and arachidonic acids (18:1 and 20:4) content, and a reversed ratio of 18:0 to 18:1 fatty acids. The differences in fatty acid composition among these species could be a reflection of species or diet effects^14^.

This study described in detail the extent, size, number and content of LDs in bovine luteal tissue and steroidogenic cells. The study also examined the increases in LD-associated proteins and lipids that occur during the follicle to luteal transition. Further research examining the impact of obesity, undernutrition and polycystic ovary syndrome on luteal LDs may provide insights into mechanisms of infertility and disorders of steroidogenesis. Additional studies examining the LDs in theca and granulosa cells and the mechanisms controlling the onset of LD presence in luteal cells could reveal their role in steroid production. Luteal LDs likely play a critical role in progesterone production by storing cholesteryl esters and interacting with mitochondria and endoplasmic reticulum to optimize steroid synthesis and provide substrates for energy production.

## Materials and methods

### Animals

Non-lactating beef cows between 2–6 years in age (n = 15) of composite breeding [75% Red Angus and 25% MARC III (1/4 Angus, 1/4 Hereford, 1/4 Pinzgauer, 1/4 Red Poll)] from the beef physiology herd at the University of Nebraska Agricultural Research and Development Center were synchronized using a modified 7-day CO-Synch protocol that utilizes GnRH and a controlled internal drug release device (CIDR; 1.38 g progesterone, Zoetis, Florham Park, NJ) to synchronize animal estrous cycles. The CIDR is removed after 7 days and animals administered prostaglandin F2α (25 mg; Lutalyse, i.m., Zoetis Inc., Kalamazoo, MI). A second GnRH injection was administered 36 h after prostaglandin F2α and 3 or 10 days post-GnRH injection, 3–5 cows were subjected to a bilateral ovariectomy. Ovaries were removed via a right-flank approach paralumbar fossa laparotomy, to avoid the rumen and prevent internal hemorrhaging, as previously detailed^49,50^. Luteal tissue was prepared for microscopy and remaining tissue was snap frozen using liquid N_2_. Day 10 corpora lutea weighed significantly more than day 3 corpora lutea (4.7 ± 0.46 vs. 2.8 ± 0.65 g, respectively). However, in all other measures examined there were no significant differences. Therefore, data from both day 3 and day 10 were pooled to provide additional statistical power and narrow the confidence intervals associated with the progesterone-secreting bovine corpora lutea. The University of Nebraska—Lincoln IACUC committee preapproved all animal procedures and all procedures were performed in accordance with the guidelines and regulations of the approved IACUC protocols and with Animal Research: Reporting in vivo Experiments^51^.

### LD staining in luteal tissue

Tissue sections were frozen in OCT (Tissue-Tek) and transported on dry ice. Frozen samples were kept at − 80 °C until sectioning using a Leica CM3050S instrument and attached to silane-coated slides before fixation in 10% phosphate-buffered formalin for 1 h. Select fixed slides were stained with oil red O and counterstained with Harris’ hemotoxin using an automated slide staining set up at the University of Nebraska Medical Center Tissue Sciences Facility. Slides were scanned at 40 × using Ventana’s Coreo Au Slide Scanner. Images were analyzed by Definiens Tissue Studio (Munich, Germany) to quantify nuclei number and area occupied by oil red O.

Coronal sections (through the stomata) of luteal tissue were fixed in 3% (w/v) paraformaldehyde and 0.2% glutaraldehyde in PBS, pH 7.4, post-fixed in 2% OsO_4_, resin-embedded, and ultra-thin sectioned for electron microscopy. Transmission electron microscopy images were captured using a Hitachi H7500 at the University of Nebraska Lincoln Center for Biotechnology. Three images (magnification: 8,000×) from luteal tissue from each animal were used for quantification of LD number and area using ImageJ^52^.

Additional fixed luteal tissue slides were immunolabeled for a marker of steroidogenic cells, 3 beta-hydroxysteroid dehydrogenase/Delta 5– > 4-isomerase type 1, and incubated at 4 °C for 24 h. Slides were washed 3 times with 0.1% Tween in PBS to remove unbound antibody and counterstained using appropriate secondary antibodies, BODIPY 493/503 and DAPI. Slides were washed again and mounted to glass microscope slides using Fluoromount-G (Electron Microscopy Sciences) and stored at − 20 °C until imaging. Additional antibody and staining information are available in Table 1.

**Table 1:** Characteristics of antibodies and reagents used for western blotting and microscopy.

**Antibody name**	**Dilution**	**Source**	**Supplier (distributor, town, country)**	**Cat. No**
Perilipin 1	1:1,000	Rabbit pAB	Sigma Life Science (St. Louis, Missouri, USA)	HPA024299
Perilipin 2	1:1,000	Rabbit pAB	Novus Biologicals (Centennial, Colorado, USA)	NB110-40877
Perilipin 3	1:1,000	Rabbit pAB	Abcam (Cambridge, United Kingdom)	47638
Perilipin 5	1:1,000	Guinea Pig pAB	Progen Biotechnik (Heidelberg, Germany)	GP31
Adipose triglyceride lipase	1:1,000	Rabbit pAB	Cell Signaling (Danvers, Massachusetts, USA)	2138S
Abhydrolase domain containing 5	1:1,000	Mouse mAB	Novus Biologicals	H00051099-M01
Hormone Sensitive Lipase	1:1,000	Rabbit pAB	Cell Signaling	4107S
Sterol O-acyltransferase 1	1:1,000	Rabbit pAB	Abcam	ab72229
Glyceraldehyde 3-phosphate dehydrogenase	1:1,000	Mouse mAB	EMD Millipore (Burlington, Massachusetts, USA)	MAB374
Aromatase	1:200	Rabbit pAB	Abcam	ab80206
3 beta-hydroxysteroid dehydrogenase	1:1,000	Rabbit mAB	A gift from Dr. Ian Mason	
β-actin	1:5,000	Mouse mAB	Sigma Life Science	A5441
α-rabbit HRP-linked	1:10,000		Jackson Research (West Grove, PA, USA)	111-035-003
α-guinea pig HRP-linked	1:10,000		Jackson Laboratory	106-035-003
α-mouse HRP-linked	1:10,000		Jackson Laboratory	115–035-205
α-rabbit Alexa Fluor 594	1:500		Invitrogen (Carlsbad, CA, USA)	A-11012
Mitotracker Deep Red FM	500 nM		Thermo Fisher (Carlsbad, CA, USA)	M22426
Phalloidin	1:500		Thermo Fisher	A34055
BODIPY 493/503	20 µM		Thermo Fisher	D3922
BODIPY FL C_12_	1 µM		Thermo Fisher	D3822
TopFluor Cholesterol	5 µM		Avanti Polar Lipids (Alabaster, Alabama, USA)	810255
DAPI^a^	300 nM		Thermo Fisher	D1306

^a^4′,6-diamidino-2-phenylindole (DAPI).

### Isolation of granulosa, theca, large luteal, and small luteal cells

For luteal cell preparations, bovine ovaries were collected from a local abattoir during early pregnancy (fetal crown-rump length < 15 cm)^53^, dissociated with collagenase, and centrifugal elutriation was performed to prepare enriched preparations of small and large luteal cells, as described previously^54,55^. The average purity of small luteal cells was > 90% and large luteal cells was 60% ± 10%.

Follicular granulosa and theca cell pools were prepared from bovine ovaries with large follicles (> 8 mm diameter) collected from a local abattoir. Ovaries were visually assessed for healthy-appearing follicles and the corresponding follicular fluid was confirmed to be > 0.8 mL and clear of blood and gross cellular contamination^56^. Follicular granulosa cells from approximately 10 follicles were suspended in DMEM/F12 culture media. After the granulosa cells were removed, the theca interna was removed with fine forceps. Granulosa cells were washed by centrifugation three times at 150 rcf for 5–10 min and filtration through a 70 µm nylon mesh. The theca interna were suspended in collagenase 2 (103 IU/mL, Atlanta Biologicals) in DMEM/F12 and dispersed using constant agitation at 37 °C for 1 h. Dispersed theca cells were removed from the undigested tissue by filtration through a 70 µm mesh then washed by centrifugation three times at 150 rcf for 5–10 min.

### Microarray

Bovine gene expression arrays from NCBI GEO repository (GSE83524) were mined to analyze expression of expression of LD components in freshly isolated bovine granulosa (n = 4) and theca (n = 3) cells from large follicles and from purified preparations of bovine small (n = 3) and large (n = 3) luteal cells from mature corpora lutea. Details of the isolation and analysis were previously published^29^.

### LD isolation from tissue

Bovine luteal, adipose, cardiac, hepatic and pulmonary tissues were obtained from a local abattoir. For luteal tissue, bovine ovaries were collected from a local abattoir during early pregnancy (fetal crown-rump length < 15 cm)^53^. The tissues (~ 2.5 g) were washed thoroughly in TE buffer (10 mM Tris, 1 mM EDTA, pH 7.4). Tissue was minced in 10 mL tissue homogenate buffer (60% sucrose w/v in TE buffer containing protease and phosphatase inhibitor cocktail) and homogenized with a Teflon Dounce homogenizer in a glass vessel. The post-nuclear supernatant fraction was obtained after centrifugation at 2000 rcf for 10 min. The supernatant was loaded into a 30 mL ultracentrifuge tube and overlaid sequentially with 40%, 25%, 10%, and 0% sucrose w/v in TE buffer containing protease and phosphatase inhibitor cocktails. Samples were centrifuged at 110,000 rcf for 30 min at 4 °C with no brake in a Beckman Coulter Avanti J-20 XP ultracentrifuge using an SW 32 Ti rotor. LDs concentrated in a band at the top of the gradient were harvested and concentrated by centrifugation at 2000 rcf for 10 min at 4 °C. The protocol was modified from^32,57^.

### Western blots

Western blots were performed as previously described^54^ with the antibodies and reagents described in Table 1.

### Lipidomics

Lipids from luteal LDs were extracted using a standard Bligh and Dyer extraction protocol^58^ and then dried under a stream of nitrogen and sent to Avanti Polar Lipids for lipidomic profiling of free sterols, cholesteryl esters, TGs, DGs, phospholipids, and sphingolipids. The molecular species within each class were identified, quantified, and summed to report the average lipid profile of bovine luteal LDs. To provide resolution and quantitative ability beyond the mass resolution of the tandem quadrupole mass spectrometers employed, molecular species were resolved by reversed-phase liquid chromatography in the presence of class and sub-class specific internal standard compounds added to each sample. The compounds were detected by tandem multiple reaction monitoring MS/MS for mass specific fragment ions according to lipid class and molecular weight of the compound. Selectivity was further enhanced by scheduling the detection of each compound according to its elution from the high-performance liquid chromatography column, known as scheduled multiple reaction monitoring. The semi-quantization was calculated using the integrated area of each analyte’s peak, divided by the appropriate internal standard peak area, and multiplied by the internal standards known concentration. Quantification of cholesterol and cholesteryl esters were directly calculated with standards and internal standards from calibration response curves. Lipid concentrations were normalized to the corresponding protein concentration of each sample and as mol % relative to lipid class.

### High performance thin layer chromatography

For lipid analyses, cell suspensions were extracted with chloroform–methanol (1:1). High-performance thin layer chromatography was performed as previously described with modification to a single solvent system [petroleum ether (b.p. 60–70 °C)-ethyl ether-acetic acid (45:5:0.5)]^59^. The images were analyzed using UVP Vision Works LS software by calculating the area under the curve after lane-specific straight line background correction. A mixture of the following standard lipids was co-chromatographed: cholesterol, trioleate glyceride, cholesteryl palmitate, and oleic acid. Preliminary analyses were completed to establish the linearity of detection for each lipid class to ensure that lipids did not exceed the linear range for quantitation. For every plate of cellular lipids, five lanes of varying amounts of lipid standards were simultaneously run to generate standard curves for quantitation. The amount of each cellular lipid was expressed as µg lipid/mg cell protein or µg lipid/mg initial tissue mass. The protocol was adapted from^60^.

### Confocal microscopy and analysis

To characterize LDs in small and large bovine luteal cells, thin-layer cell preparations for confocal microscopy of enriched small luteal cells > 90% and large luteal cells (purity range: 50–90%; mean: 65.3 ± 14.6) were prepared using a Cytofuge 2 (Beckman Coulter). Cells were then fixed with 10% neutral buffered formalin at 4 °C for 30 min. Cells were stained with BODIPY 493/503 and phalloidin (Table 1) for 1 h at room temperature and mounted using Fluoromount-G.

To determine the colocalization of BODIPY C12 and TopFluor Cholesterol in luteal LDs, **e**nriched small (5 E 04 cells/cm^2^) or large (2 E 04 cells/cm^2^) luteal cell cultures were seeded onto **s**terile No. 1 glass coverslips (22 × 22 mm). Cells were treated with TopFluor Cholesterol and BODIPY C12 for 48 h to allow incorporation in LDs. Cells were then incubated in Mitotracker Deep Red for 45 min to label mitochondria (Table 1). Cells were then fixed with 4% paraformaldehyde at 4 °C for 30 min and mounted using Fluoromount-G. Images were collected using a Zeiss confocal microscope equipped with a 60 × oil immersion objective (1.4 N.A) and acquisition image size of 512 × 512 pixel (33.3 µm × 33.3 µm). Cells were randomly selected from each slide and 0.33 μm slice z-stacked images were generated from bottom to top of each cell. A 3-dimensional image of each cell was created, and the area of individual cells was determined using Zen software. Images were converted to maximum intensity projections and processed utilizing ImageJ (National Institutes of Health) analysis software. LD size and number were quantified with ImageJ using AnalyzeParticles function in threshold images, with size (square pixel) setting from 0.1 to 100 and circularity from 0 to 1. Outputs were then converted into microns. For colocalization of BODIPY C12 and TopFluor Cholesterol, z-stack images were analyzed in Image J using the JACoP plugin as previously described^61^.

### Statistical analysis

All data are presented as means ± SEM. Data was evaluated for normal distribution and log transformed if necessary. Specifics of statistical testing are described in the relevant figure legends. Statistical analysis was performed using GraphPad Prism (GraphPad Software, Inc) except for the microarray data which was analyzed as previously described^29^.

## Supplementary information

Supplementary file1

## Data Availability

Lipidomics data: Metabolomics Workbench, ST001286, https://www.metabolomicsworkbench.org/data/DRCCMetadata.php?Mode=Study&StudyID=ST001286. Microarray data: NCBI GEO repository, GSE83524, https://www.ncbi.nlm.nih.gov/geo/query/acc.cgi?acc=GSE83524
