# Supplementary material for: Formation and characterization of lipid droplets of the bovine corpus luteum
Source: Sci Rep. 2020 Jul 9;10:11287. doi: 10.1038/s41598-020-68091-2 (PMC7347867; doi:10.1038/s41598-020-68091-2)

Supplemental Figure 1
Formation and Characterization of Lipid Droplets of the Bovine Corpus Luteum.
Talbott, H.A., Plewes, M.R., Krause, C., Hou, H., Zhang, P, Rizzo, W.R., Wood, J.R., Andrea S. Cupp, A.S., and Davis, J.S,.; Full Western blotting images of Figure 2

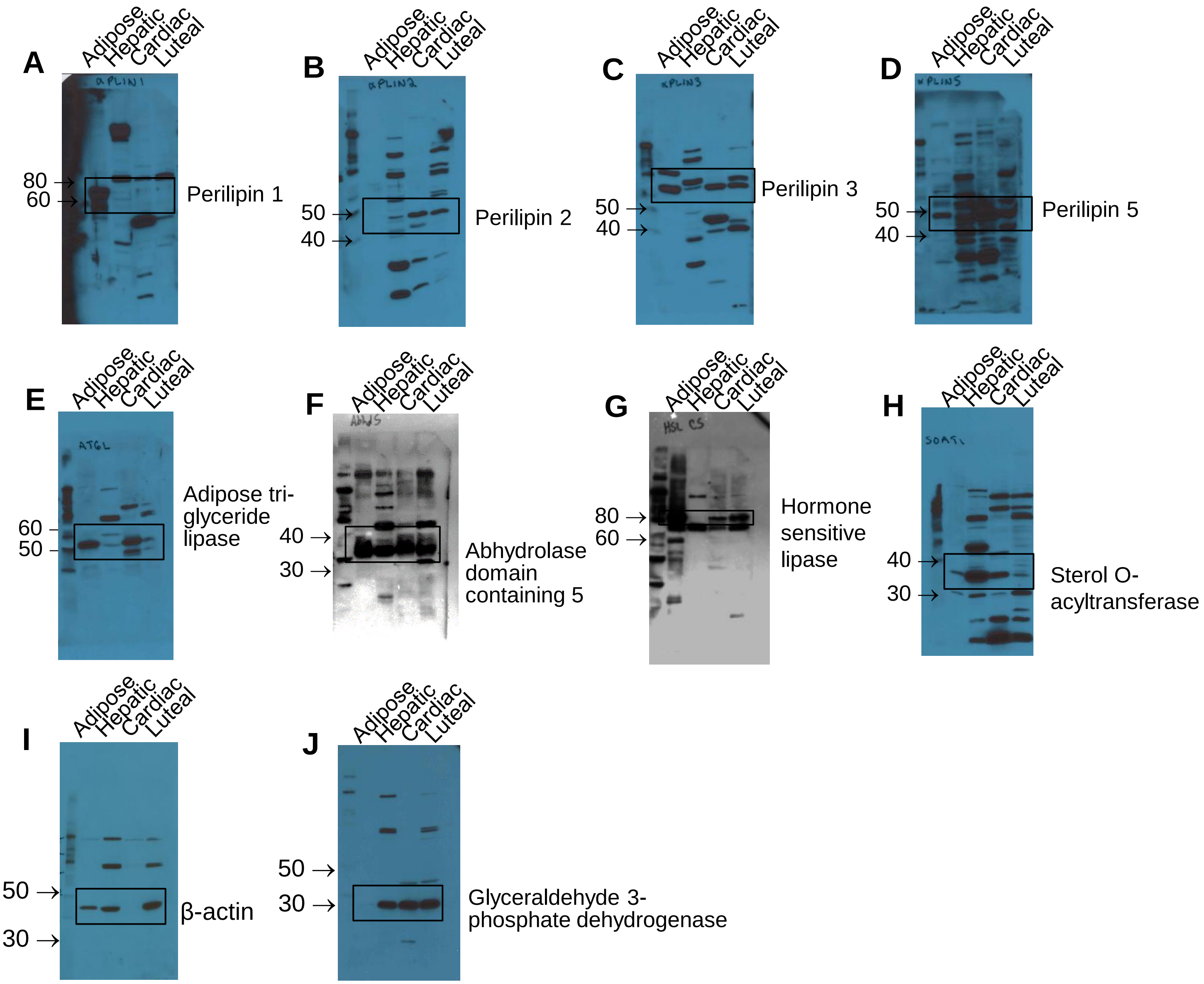

Supplemental Figure 2

Formation and Characterization of Lipid Droplets of the Bovine Corpus Luteum**.**

Talbott, H.A., Plewes, M.R., Krause, C., Hou, H., Zhang, P, Rizzo, W.R., Wood, J.R., Andrea S. Cupp, A.S., and Davis, J.S,.; Full Western blotting images of Figure 3

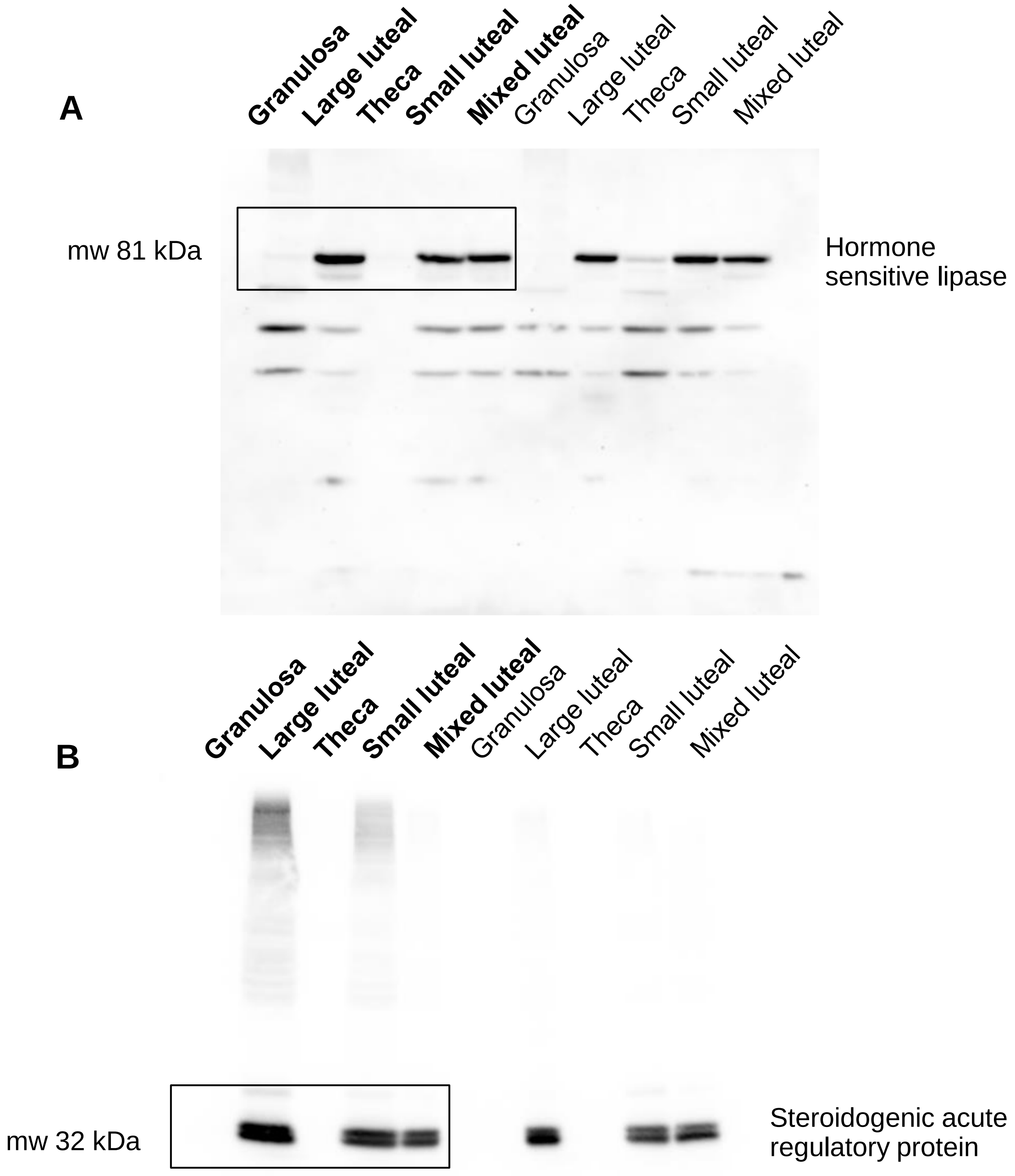

Supplemental Figure 2
Formation and Characterization of Lipid Droplets of the Bovine Corpus Luteum.
Talbott, H.A., Plewes, M.R., Krause, C., Hou, H., Zhang, P, Rizzo, W.R., Wood, J.R., Andrea S. Cupp, A.S., and Davis, J.S,.; Full Western blotting images of Figure 3

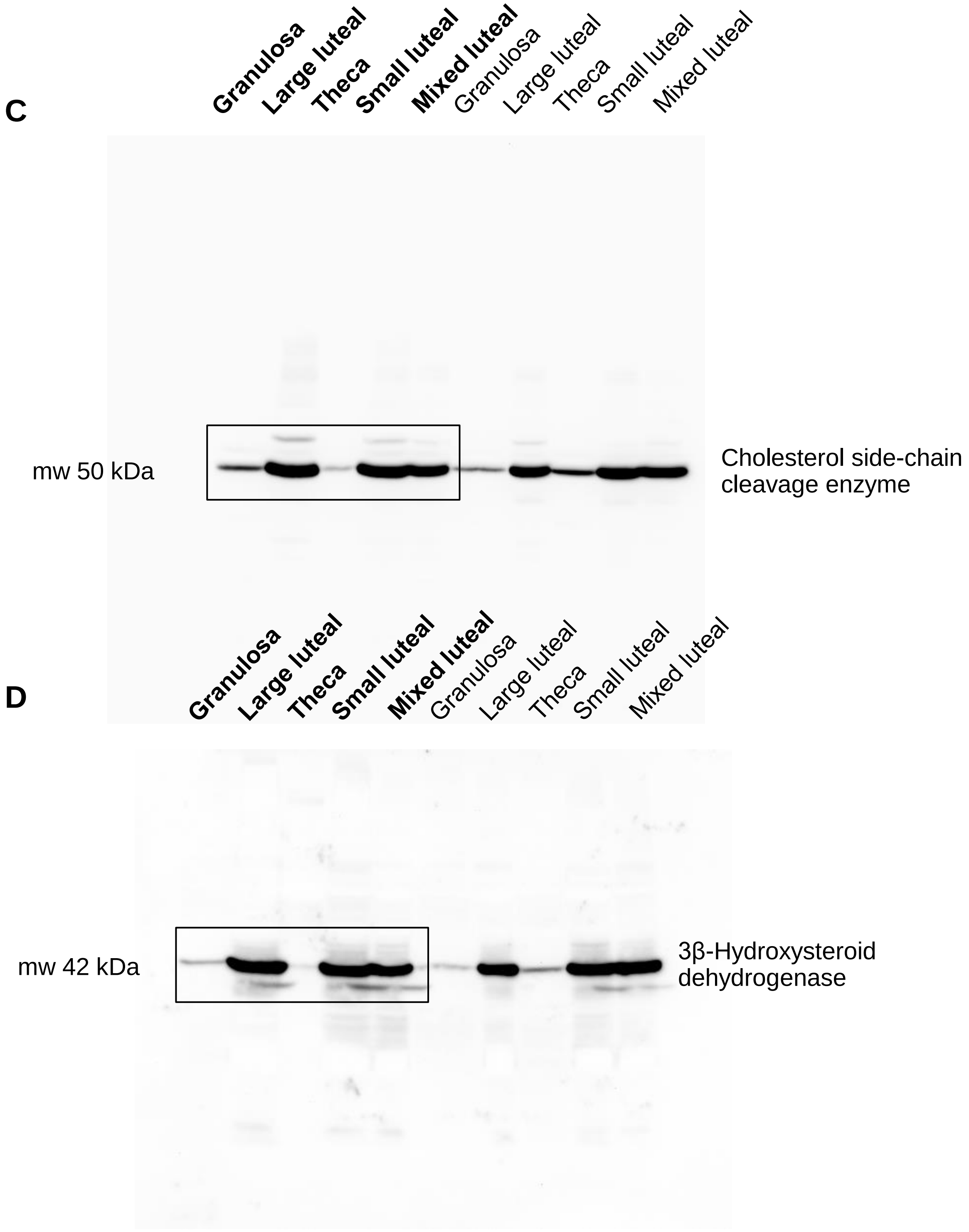

Supplemental Figure 2
Formation and Characterization of Lipid Droplets of the Bovine Corpus Luteum**.**
Talbott, H.A., Plewes, M.R., Krause, C., Hou, H., Zhang, P, Rizzo, W.R., Wood, J.R., Andrea S. Cupp, A.S., and Davis, J.S,.; Full Western blotting images of Figure 3

E

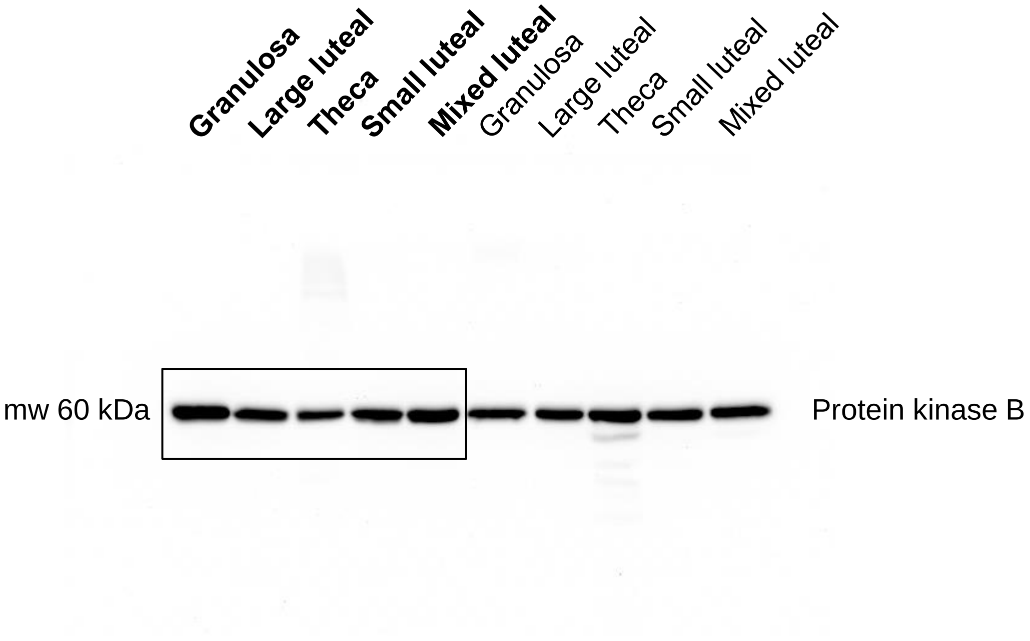

F

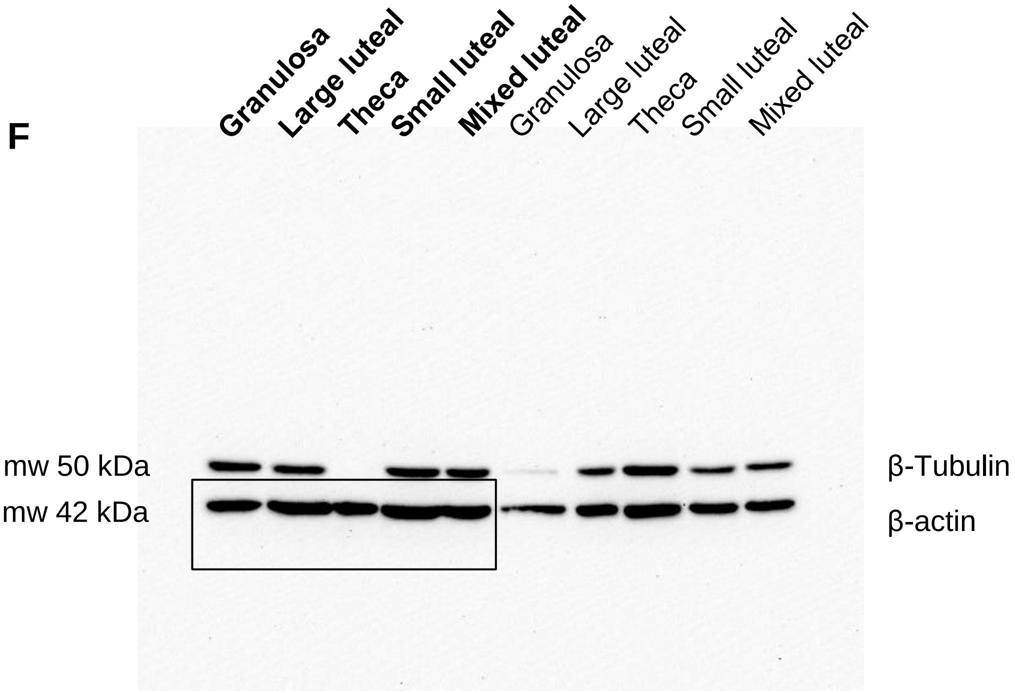

Supplement: Supplementary file 1 — Supplementary file1 [file 41598_2020_68091_MOESM1_ESM.pdf]
